# Administration of ivermectin to peridomestic cattle: a promising approach to target the residual transmission of human malaria

**DOI:** 10.1186/s12936-015-1001-z

**Published:** 2015-12-10

**Authors:** Hermann S. Pooda, Jean-Baptiste Rayaisse, Domonbabele François de Sale Hien, Thierry Lefèvre, Serge R. Yerbanga, Zakaria Bengaly, Roch K. Dabiré, Adrien M. G. Belem, Issa Sidibé, Philippe Solano, Karine Mouline

**Affiliations:** Centre International de Recherche-Développement sur l’Elevage en Zone Subhumide, Bobo-Dioulasso, Burkina Faso; Ministère des Ressources Animale/Campagne Panafricaine d’éradication de la mouche tsé-tsé et des trynaposomoses, Bobo-Dioulasso, Burkina Faso; Direction Régionale de l’Ouest de l’Institut de Recherche en Sciences de la Santé, Bobo-Dioulasso, Burkina Faso; MIVEGEC (Maladies Infectieuses et Vecteurs: Ecologie, Génétique, Evolution et Contrôle), UMR IRD 224, CNRS 5290, Université de Montpellier, 911 Av. Agropolis, Montpellier, France; Université Polytechnique de Bobo-Dioulasso, Bobo-Dioulasso, Burkina Faso; UMR INTERTRYP IRD-CIRAD, TA A 17/G, Campus International de Baillarguet, 34398 Montpellier cedex 5, France

**Keywords:** Ivermectin, Cattle, One-Health, Malaria, West Africa, *Anopheles coluzzii*, Survival, Fecundity, Infectivity

## Abstract

**Background:**

The success of current control tools in combatting malaria vectors is well established. However, sustained residual transmission of *Plasmodium* parasites persists. Mass drug administration (MDA) to humans of the endectocide ivermectin for vector control is receiving increasing attention. However, vectors feeding upon animals escape this promising approach. Zoophagy of mosquitoes sustains both the vector population and endemic population of vector-borne pathogens. Therefore, only a strategy that will combine ivermectin MDAs targeted at humans and their peridomestic animals could be successful at controlling residual malaria transmission.

**Methods:**

Burkinabé cattle have been treated with injectable therapeutic dose of ivermectin (0.2 mg/kg of body weight) to render blood meals toxic to field representative populations of *Anopheles coluzzii* carrying the *kdr* mutation. Direct skin-feeding assays were performed from 2 to 28 days after injection (DAI) and mosquitoes were followed for their survival, ability to become gravid and fecundity. Membrane feeding assays were further performed to test if an ivermectin blood meal taken at 28 DAI impacts gametocyte establishment and development in females fed with infectious blood.

**Results:**

The mosquitocidal effect of ivermectin is complete for 2 weeks after injection, whether 12 days cumulative mortalities were of 75 and 45 % the third and fourth weeks, respectively. The third week, a second ivermectin blood meal at sub-lethal concentrations further increased mortality to 100 %. Sub-lethal concentrations of ivermectin also significantly decreased egg production by surviving females, increasing further the detrimental effect of the drug on vector densities. Although females fitness was impaired by sub-lethal ivermectin blood meals, these did not diminish nor increase their susceptibility to infection.

**Conclusion:**

This study demonstrates the potential of integrated MDA of ivermectin to both human and peridomestic cattle to target vector reservoirs of residual malaria transmission. Such integration lies in ‘One-Health’ efforts being implemented around the globe, and would be especially relevant in rural communities in Africa where humans are also at risk of common zoonotic diseases.

**Electronic supplementary material:**

The online version of this article (doi:10.1186/s12936-015-1001-z) contains supplementary material, which is available to authorized users.

## Background

The success of long-lasting insecticidal nets (LLINs) and indoor residual spraying (IRS) in combatting malaria transmission by *Anopheles* mosquitoes is well established. However, transmission of *Plasmodium* parasites persists despite effective coverage being achieved with LLINs and IRS interventions. Besides the evolution of physiological resistance allowing a mosquito to survive despite direct contact with insecticides (either by target site mutations and/or metabolic resistance [1]), vectors responsible for residual transmission can exhibit specific behaviours, such as biting at unusual times, that allow them to escape the fatal exposure to LLINs or IRS [2, 3]. Insecticide avoidance, exophily, exophagy, but also zoophagy [4], are all behaviours that minimize the contact between the mosquito and the insecticides, and contribute to the build-up of reservoirs of vector populations responsible for residual transmission of diseases.

In this context, mass drug administration (MDA) of endectocidal drugs to humans for human malaria control is receiving increasing attention [5, 6]. Endectocides are drugs that have activity against endo- and ectoparasites among which ivermectin was first introduced for commercial use as an anti-parasitic drug for animal (livestock and pets) use in 1981. This molecule shares with other avermectins and mylbemicins a pharmacophore consisting on 16-membered macrocyclic lactone, and is an agonist of specific chloride ion channels (primarily glutamate-gated chloride channels). As these channels are neurotransmission inhibitors, ivermectin leads to flaccid paralysis, which culminates in the animal death [7]. Ivermectin is the only known endectocide currently approved for human use and is now massively distributed as part of pan-African programs for onchocerciasis control and lymphatic filariasis elimination [8, 9]. The broad range of invertebrates it targets includes mosquito vectors of diseases, such that ivermectin is now proposed as an additional tool to control vector-borne diseases such as malaria [5, 6, 10–15]. Hence, numerous in vitro and in vivo studies have shown that a blood meal containing ivermectin causes a significant reduction in adult female mosquito longevity, fecundity and fertility [5]. In experimental infections of malaria mosquitoes, ivermectin was also shown to inhibit *Plasmodium* sporogony [16]. Recent field-based studies have demonstrated that MDA using ivermectin can significantly reduce the survivorship of adult field-caught *Anopheles* mosquitoes [11, 12, 14, 15]. Ivermectin, thus, seems to negatively affect a series of mosquito traits (longevity, fecundity, competence to pathogens), which are keys in determining the intensity of disease transmission. In other words, ivermectin can reduce mosquito vectorial capacity. The straightforward rationale of using ivermectin MDA for vector control lies on the fact that the treated human directly delivers the toxic molecule to any human-feeding mosquito regardless of its genus, species, and possibly for a large spectrum of the behavioural resistance it might display, i.e., mosquitoes of diurnal or nocturnal activities, resting indoors or outdoors, feeding indoors or outdoors, could be targeted.

The glutamate-gated chloride channel, primary targets of ivermectin, has recently been characterized in *Anopheles gambiae*, where it is expressed in motor and sensory neurons [17]. Like for other invertebrates [18, 19], ivermectin has been shown to potentiate glutamate action in malaria mosquitoes [17]. This mode of action is distinct compared to current (and candidate) insecticides for IRS application (pyrethroids, organochlorines, organophosphates, and carbamates) and LLIN treatment (pyrethroids), making ivermectin administration a promising tool for integrated vector control and insecticide resistance management in malaria vectors.

However, vectors feeding upon animals will still escape this approach. *Anopheles* mosquitoes are able to feed on many other vertebrates than humans and secondary malaria vectors feed primarily on animals, outdoors. Because they only feed occasionally on humans these mosquitoes are poor disease vectors, but since they respond poorly to LLIN or IRS interventions, they are therefore responsible for limited but self-sustaining disease transmission [4]. Highly anthropophilic *Anopheles* species also display zoophilic and outdoor blood-feeding behaviour in response to the altered patterns of blood source availability following IRS or LLIN implementation [20]. For example, a study conducted in an area of extensive coverage with LLINs showed that whereas the anthropophilic rate (as measured with odour-baited entry traps) of *Anopheles coluzzii* was 88 %, over 50 % fed on cattle, indicating a plastic feeding strategy with a zoophilic pattern of host selection despite a stronger response to human odour. In this field population, *An. coluzzii* has an innate preference for humans but the weak accessibility of this host species, due to the use of bed nets, forces the mosquitoes to feed on cattle, an available, less preferred host [21]. Zoophagy of mosquitoes, either innate or induced by control interventions, can therefore sustain the build-up of a reservoir of vector populations responsible for the residual transmission of parasites.

Ivermectin is also widely used by veterinarian services for the control of parasites of companion animals and livestock [22]. Therefore, animal treatment with ivermectin as a supplementary tool for controlling vectors of human disease would be rather straightforward to implement and, hence, this approach has received little attention [4, 5, 23]. However, it is not yet considered as an intrinsic, mandatory part of future malaria vector control approaches using MDA of ivermectin distributed to humans. Such integration would lie in ‘One-Health’ efforts being implemented around the globe, and would be especially relevant in rural farming communities in Africa where humans are also at risk of common zoonotic diseases.

In Burkina Faso, around 77 % of the population live in rural areas [24], where people mainly rely on small family farming for their livelihoods, based on cereals and cotton cropping, livestock breeding and tree product collection. Almost each farm household owns a pair of oxen dedicated to field labour. The present work explores the possibility of integrated ivermectin MDA measures, which would benefit humans and animals in rural areas of Burkina Faso. Other studies have dealt with the impact of ivermectin on life history traits of *Anopheles gambiae s.l.* fed on cattle [22, 25–27] and on their vector competence for laboratory strain of *P. falciparum* [16]. However, the present study uses a combination of sympatric, recently established mosquito colonies, local calves and field-collected strains of *P. falciparum*, with the aim of being as relevant as possible in establishing the proof of concept that ivermectin-treated cattle could be used as an additional tool to circumvent residual malaria transmission in rural Africa.

## Methods

### Mosquito colony

The *An. coluzzii* colony hosted at the Institut de Recherche en Sciences de la Santé (IRSS), Bobo Dioulasso, Burkina Faso insectary facility was used. This colony was established in 2008 and repeatedly replenished with F1 from wild-caught mosquito females collected in Kou Valley (11°23′14″N, 4°24′42″W), 30 km from Bobo-Dioulasso, southwestern Burkina Faso (West Africa), and identified for their species status by routine PCR [28]. Potential contamination of the colony by other *Anopheles* species was routinely checked using the same technique. Mosquitoes were maintained under the standard conditions of 27 ± 2 °C, 75 ± 5 % relative humidity and 12 h/12 h day/night cycle. Larvae were reared at low densities in plastic trays in tap water and fed ad libitum with commercial alevin food [Tetramin^®^ Baby Fish Food (Tetrawerke, Melle, Germany)]. Pupae were collected in cups and placed in 30 × 30 × 30 cm cages. Newly emerged adults were allowed to feed for three to 5 days on 5 % glucose solution then starved for 16–18 h before blood feeding on cattle.

Thirty-two females were randomly chosen in the colony to characterize their physiological resistance status by PCR following [29] for the *kdr* mutation and for *ace1* [30]. Eight females carried the mutated *kdr* allele, and all carried the wild alleles of the *ace*-*1* gene. The *kdr* mutation checked for in the colony refers to the West-African *kdr* mutation (i.e. kdr-W or L1041F), as the *kdr*-*E* (or L1041S, first evidenced in East-Africa) has not yet been reported in the area of Bama for *An. coluzzii* [31].

### Cattle hosts

Four bull calves (mean weight = 91 ± 24 kg) of the local Metis breed (obtained from cross breedings between Fulani zebus and Baoulé bulls) were used as hosts for *Anopheles* blood feeding. Upon their arrival to the Centre International de Recherche-Développement sur l’Elevage en zones Subhumides (CIRDES) stable facilities, (i.e., 1 month before the start of experiments), the calves were systematically treated with therapeutic doses of aceturate diminazene and albendazole to, respectively, cure potential trypanosomiasis (endemic in this area) and gastro-intestinal infestation with endoparasites, which could affect their well-being. During the experiment, calves were fed with a diet made of straw and cotton oil cake and provided with water ad libitum. They were maintained in the stable, protected by a net to avoid any insect disturbance, and checked every other day by a veterinarian to ensure their well-being.

### Ivermectin treatment

Two calves were randomly chosen to receive a subcutaneous injection of ivermectin (IVOMEC D^®^) at the therapeutic dose of 0.2 mg/kg of body weight (treated calves, A and C), while the two other calves received no treatment (controls, B and D).

### Blood feeding

**Fig. 1:**
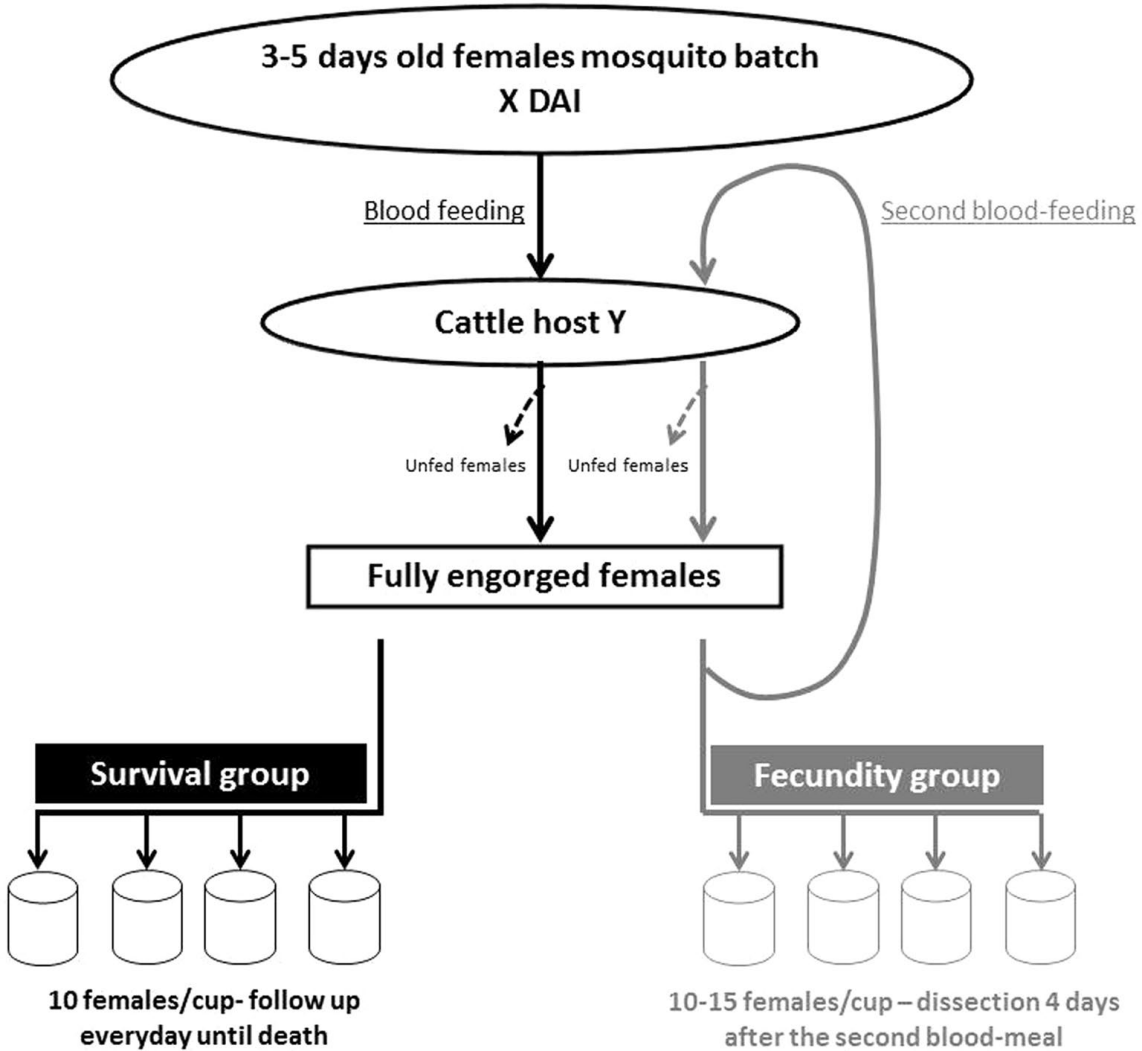
Diagrammatic representation of the sequential events forming the study’s experimental design. *X* represents either 0, 2, 7, 14 or 28 DAI whether *Y* is for the calf A, B, C or D

Three to five days old mosquitoes were randomly introduced into 16 plastic cups covered with nets (n = 30 mosquitoes per cup). Four plastic cups were randomly assigned to each control and treated calf and disposed on the sides of the calves, where they were held using a rubber strap arranged around the animals’ abdomen. Calves were restrained using ropes to avoid rough movements and scratching. Mosquitoes were allowed to feed for 15 min, after which only fully engorged females were transferred in maintaining cups or cages, for survival and fecundity evaluation, respectively (Fig. 1 for a diagrammatic representation of the experimental set-up). Blood feeding of mosquitoes occurred in six instances: once before treatment and at different times points after ivermectin treatment, taking into account previous experiments (Additional files 1 and 2), and already published plasmatic pharmacokinetics of the molecule in cattle [32]: i.e, at 2, 7,14, 21, and 28 days after the injection (DAI). Different batches of mosquitoes were used for each blood-feeding episode. The percentage of blood-fed mosquitoes was similar between the seven batches (i.e. 95 %), for each treatment and each calf.

### Life history traits of mosquitoes fed on treated and control cattle

#### Survival

Fully engorged females were randomly distributed, maintained in paper cups (cup volume was 455 cu). Four cups were used per calf with ten mosquitoes per cup (Fig. 1) and provided every day with cotton balls soaked in 2.5 % glucose solution. Mortality was recorded every day from the day of blood feeding until all mosquitoes died.

#### Reproduction

In *An. gambiae s.l.*, the first blood meal is often used to compensate nutritional deficiencies carried over from larval stages instead of developing ovarian follicles [33]. This was also observed in the IRSS’ *An. coluzzii* colony, for which only a small proportion of females developed eggs after only one blood meal [34]. For this reason and in line with previous studies [35], the number of eggs produced after two consecutive blood meals was considered as more representative of mosquito fecundity. Hence, 4 days after a first blood meal, female mosquitoes took a second blood meal on the same host than the first (Fig. 1). Dissections of the ovaries were performed 4 days afterwards, when the second blood meal was entirely digested. The second blood meal success was similar to the first (i.e. 90 %) and only females that had actually taken two blood meals were considered. Ovaries were extracted from the abdomen and dissected in a drop of Phosphate Buffered Saline (PBS) to release the eggs, which were counted under a binocular (40×, Leica S6D). The number of females carrying developed eggs (i.e., egg prevalence) and the number of mature eggs (i.e., Christopher stage V) developed by a female were considered as proxies of their fecundity [33]. As for survival experiments, for each mosquito lot (see above), fully engorged females were randomly distributed and maintained in paper cups (four cups per calf, ten to 15 mosquitoes per cage) until the dissection.

### Effect of a sub-lethal dose of ivermectin on the sporogonic development of *Plasmodium falciparum*

Experimental infections of *An. coluzzii* females with *P. falciparum* gametocytes were processed by membrane feeding as previously described [36, 37]. Thick blood smears from 5–11 years old children from the village of Dandé (11°34′48″ N, 4°33′36″ W) were examined using light microscopy to identify gametocyte carriers. Gametocyte density was evaluated against 1000 white blood cells (WBC) and expressed per µl, assuming the canonical number of 8000 WBC/µl of blood. A gametocyte carrier with 64 gametocytes/µl was selected for the experiment. Five ml of venous blood were collected and distributed in each membrane feeder and maintained at 37 °C by circulating heated water. In nature, it is likely sequentially that a mosquito will absorb sub-lethal doses of ivermectin from a cattle host and, 2–4 days later, an infectious blood meal from a human host. For ethical considerations, only this “prophylactic” combination was tested (i.e. whether an ivermectin containing bloodmeal would protect the mosquito from a subsequent infection by *Plasmodium falciparum*), since other combinations (i.e. the ivermectin containing blood meal given the same day or 3–4 days after the infectious one (the later being the “therapeutic” combination)) would require potentially infectious mosquitoes to be transported from IRSS to CIRDES. At 28 DAI, batches of mosquitoes that had already taken their first blood meal on treated and control cattle 4 days before (see above for blood feeding processing) were infected. For that, mosquitoes were disposed under the feeders and allowed to feed for 30 min. Only fully engorged mosquitoes were followed up for parasite development, and for this purpose, were provided ad libitum 5 % glucose solution for 8 days after membrane feeding. Mosquito midguts were then dissected in a drop of 0.5 % mercurochrome, mounted on a slide, covered with a coverslip and examined under a light microscope (20×, Leica ICC 50) to detect and count the oocysts.

### Statistics

All statistical analysis were performed using the software R version 3.1.3 GUI 1.65.

### Mosquito survival

Kaplan–Meier survival estimates were calculated to investigate whether females’ longevities were affected by a blood meal taken on cattle at different DAI of a therapeutic dose of ivermectin. Uncensored data were used, as all mosquitoes were dead by the end of the experiment. The effects of the ivermectin treatment, the time after injection and their interaction were further tested using Cox proportional hazards model. Because the blood from different cattle may represent different nutritive values, the effect of cattle on female survival was evaluated for each DAI and within each treatment status (i.e., ivermectin-treated or control). This cattle effect on mosquito survival was also assessed for the four cattle before the ivermectin was injected (0 DAI) in order to ensure that any difference in mosquito survival was due, at least in part, to the ivermectin treatment.

### Mosquito reproduction

First, the effect of the ivermectin treatment, the DAI and their interaction on the probability that a female will become gravid was examinated by fitting logistic regression models (generalized linear modelling with binomial errors and logit link function). Second, only females that were gravid were subsequently considered and regression models (generalized linear modelling with quasipoisson errors and logit link function) were used to examine the effect of ivermectin treatment, the DAI and their interaction on the number of developed eggs. Cattle effect was also examined as described above.

### *Plasmodium* sporogonic development

The impact of sub-lethal dose of ivermectin within a first blood meal taken at 28 DAI on the infectivity of *P. falciparum* parasites absorbed during a second blood meal (i.e., infection prevalence) was tested using generalized linear models with binomial errors and logit link function. Only females that were infected were subsequently considered to examine whether the first blood meal had an impact on the number of oocysts developed by infected females (i.e., infection intensity) through generalized linear models (quasipoisson errors and logit function). As for survival and fecundity, the cattle effect has been examined as previously described.

For all the analysis, stepwise simplification of models was performed where non-significant terms were sequentially removed to produce the minimal model with the best explanatory power [38]. When needed, analysis were followed by post hoc tests procedures (multcomp package) to compare the levels of significant factors. Before the analysis, the constancy of variance between datasets was checked using the Fligner–Killeen test. When needed, a variable transformation was performed to meet this constancy.

## Results

### Survival

**Fig. 2:**
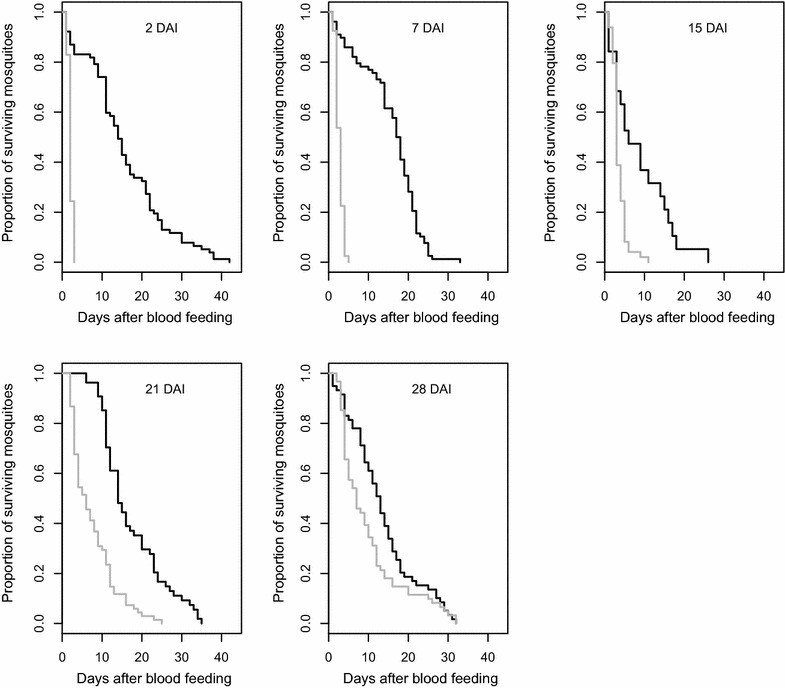
Kaplan–Meier estimates of *Anopheles coluzzii* survival when fed on treated and control cattle at different days after injection (DAI) of 200 µg/kg of ivermectin. *Black lines* mosquitoes fed on control calves; *grey lines* mosquitoes fed on treated calves

Experiments investigating the toxic effect of a blood meal taken from ivermectin-treated cattle included 960 females followed after their blood meal and until their death. For the females fed on cattle before injection of ivermectin, the Cox proportional hazards model revealed no significant effect of cattle identity on survival (χ^2^_3_ = 1.42, *p* = 0.69), which was on average 17.77 ± 1.52 days, 19.80 ± 0.88, 20.14 ± 1.22, and 21.32 ± 0.73 days. Analysis of mosquito survival after ivermectin injection showed a significant effect of the treatment, DAI and the interaction between these two factors (Table 1). This significant treatment × DAI interaction indicates a decrease in the negative impact of ivermectin on mosquito survival overtime (Fig. 2). The reduction of mosquito survival is significant and equal to 86.7 % at 2 DAI, 82.63 % at 7 DAI, and 54.35 % at 21 DAI. At 28 DAI, the mean survival is reduced by 23.48 %, but this tendency is only marginally significant (Cox proportional hazards models for 28 DAI, treatment effect, χ^2^_1_ = 3.76, *p* = 0.052). At 15 DAI, lower than usual mean survivals of 5.90 ± 1.52 and 12.75 ± 2.80 days were observed for the mosquitoes fed on, respectively, the control calves B and D, which led to underestimates of the ivermectin effect at this time period. In an additional experiment, mosquitoes fed on control calves at 15 DAI had a usual mean survival of 16.6 ± 3.85 days (Additional file 2), allowing to estimate that feeding on treated calves 15 days after injection leads to a 77 % reduction in average of the mean survival time. For the mosquitoes fed both at 21 DAI and 28 DAI, significant differences appear between the batches fed on the treated calves A and C (mean survival of females fed on cattle A and C at 21 DAI = 5.34 ± 0.78 and 10.33 ± 0.95, respectively, Cox proportional hazards models, cattle effect for this sub-set, χ^2^_1_ = 11.35, *p* < 0.001; mean survival of females fed on cattle A and C at 28 DAI = 5.63 ± 0.55 and 15.03 ± 1.75, respectively, Cox proportional hazards models, cattle effect for this sub-set, χ^2^_1_ = 25.53, *p* < 0.001). At 28 DAI, the effect of the treated cattle C on mosquito survival was no more different from the controls (comparison of mean survival of mosquitoes fed on treated calf C and control calves B and D, *p* > 0.5 for both comparisons), but differed from cattle A (*p* < 0.001). As complementary information to better apprehend the consequences of ivermectin treatment in terms of reduction of vectors densities or sporogony, we also considered the time to 100 % mortality for each DAI. At 2 DAI, 100 % of the mosquitoes would die in 2.87 ± 0.35 days, in 4 ± 0.53, 5.5 ± 3.07, 17 ± 4.24 and in 19.5 ± 11.61 days at seven, 15, 21 and 28 DAI, respectively. By comparison, 100 % mortality for the control treatment was achieved in 32.12 ± 5.74, 24.37 ± 3.99, 24 ± 4.45, 27.87 ± 5.59 and 27.12 ± 5.40 at two, seven, 15, 21 and 28 DAI, respectively. For the 15 DAI time point, values from supplemental data were taken (see Additional files 1 and 2).

**Table 1:** Effects of ivermectin treatment, the DAI and their interaction on female *An. coluzzii* survival

Source	*DF*	χ^2^	*p* value
Treatment	1	211.55	<0.0001*
DAI	4	107.97	<0.0001*
Treatment × DAI	4	167.87	<0.0001*

*DAI* days after injection

* Significant effect of parameters or interactions (*p* < 0.05)

The effect of an additional blood meal taken on treated cattle was further investigated for its impact on the survival of vector mosquitoes (Additional files 1, 2 and 3). The significant treatment × DAI × number of blood meals is due to the fact that the impact of a second blood meal varies with the time elapsed since ivermectin injection: while it had no effect at one, seven and 15 DAI, it further decreased mosquito survival at 21 DAI and was beneficial (i.e., allows a higher survival than a single blood meal) at 31 and 38 DAI (Additional file 3).

### Fecundity

**Fig. 3:**
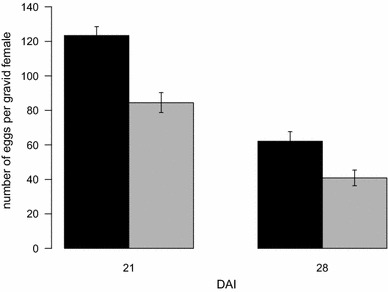
Number of eggs developed by gravid females *Anopheles coluzzii* fed on treated and control cattle at 21 and 28 days after injection of 200 µg/kg ivermectin. *Black bar* mosquitoes fed on control calves, *grey bar* mosquitoes fed on treated calves. *Error bars* are standard errors

Five hundred and forty-five mosquito females were analysed to investigate the effect of ivermectin treatment on their fecundity. The number of females that did not develop eggs was very low for females fed on cattle before ivermectin injection (i.e., three out of 92 females), but because these females were all from the batch fed on cattle A, a significant effect of the cattle host on female egg prevalence was found (χ^2^_3_ = 10.93, *p* = 0.012). However, the analysis further showed that the cattle host blood did not impact the number of eggs developed by the females (calf A 69.85 ± 11.13, calf B 89.73 ± 6.54, calf C 85.35 ± 7.46, calf D 96.37 ± 7.61, χ^2^_3_ = 18.7, *p* = 0.70). After ivermectin injection, only females fed on cattle at 7, 21 and 28 DAI could be analysed due to the absence of surviving mosquitoes 4 days after the second blood feeding in the other mosquito batches. A significant influence of the treatment (χ^2^_1_ = 14.92, *p* < 0.001), the DAI (χ^2^_1_ = 45.40, *p* < 0.001) and their interaction (χ^2^_1_ = 20.15, *p* < 0.001) was found on the probability of a female mosquito to become gravid. The treatment × DAI interaction was due to the fact that treated females fed at 7 DAI on treated cattle had a significant, lower probability of becoming gravid by comparison to their counterparts fed on control cattle (10 % (n = 10 females) vs. 89 % (n = 88 females), χ^2^_1_ = 28.69, *p* < 0.001), whereas the treatment effect was non-significant or marginally significant for mosquitoes fed at 21 and 28 DAI (χ^2^_1_ = 1.77, *p* = 0.18 and χ^2^_1_ = 0.66, *p* = 0.064, respectively). The effects of ivermectin treatment, the DAI and their interaction on the number of eggs developed by gravid females were further investigated by considering the subset of females that had developed at least one egg. Because a single female developed her eggs in the batch fed on treated cattle at 7 DAI, this batch was excluded from the analysis. Hence, the analysis was performed on the 21 DAI and 28 DAI batches only after a square-root transformation of the number of eggs per female. The model revealed no significant interaction between the treatment and the DAI, which was removed (χ^2^_1_ = 1.49, *p* = 0.37). The minimal model (Table 2) revealed a significant effect of the treatment and the DAI on the number of eggs developed by *An. coluzzii* females. The DAI effect is due to the variability between the batches for the mean number of eggs developed by the females, whether the treatment effect remains constant between 21 and 28 DAI (33 and 36 % reduction of the total number of eggs, respectively, Fig. 3). For the subset of mosquitoes fed on control or treated calves, there was no cattle effect for the number of gravid females, or for the number of eggs developed by gravid females (cattle effect for egg prevalence in control and treated sub-sets, respectively: χ^2^_1_ = 0.044, *p* = 0.50; χ^2^_1_ = 0.28, *p* = 0.31; cattle effect for the number of developed eggs in control and treated sub-sets, respectively: χ^2^_1_ = 32.47, *p* = 0.18; χ^2^_1_ = 38.53, *p* = 0.12).

**Table 2:** Effects of ivermectin treatment and of the DAI on the eggs number of *An. coluzzii*

Source	*DF*	χ^2^	*p* value
Treatment	1	48.57	<0.001*
DAI	1	142.77	<0.001*

* Significant effect of parameters or interactions (*p* < 0.05)

### Infection with *Plasmodium falciparum*

The experiment included 168 females that had already taken a first blood meal on cattle hosts either treated or non-treated at 28 DAI, and maintained with 5 % glucose solution after their infectious blood meal. Generalized linear models did not reveal any significant impact of the first ivermectin-rich blood meal on the prevalence or intensity of *An. coluzzii* infection by *P. falciparum* parasites (Table 3). Indeed, females previously fed on control or treated cattle had equivalent infection prevalence and intensity (respectively, 0.73 ± 0.04 and 3.72 ± 0.38 for females fed on controls, and 0.73 ± 0.05 and 4.07 ± 0.56 for females fed on treated cattle).

**Table 3:** Effects of sub-lethal dose of ivermectin on the *Plasmodium falciparum* gametocytogenesis in *An. coluzzii*

Source	*DF*	χ^2^	*p* value
Treatment on infection prevalence	1	0.0003	0.98
Treatment on infection intensity	1	1.33	0.24

## Discussion

Therapeutic doses of ivermectin injected to local Burkinabé Metis cattle rendered blood meals toxic to sympatric *An. coluzii* females and reduced both survivorship and fecundity of the mosquitoes feeding on treated animals for up to 28 days. For the 2 weeks following the treatment, mean survival time of mosquitoes that fed on treated cattle was two to 3.5 days (corresponding to a time to 100 % mortality of 3–5.5 days), meaning that the great majority would die before being able to resume a new gonotrophic cycle by biting a host, achieve sporogony, and eventually transmit malaria parasites. Mosquitocidal effect of ivermectin was not complete the third and fourth week after treatment and a proportion of mosquitoes was able to survive. Yet, 100 % mortality was achieved in 17 and 19 days, a timeframe just long enough to become infectious and potentially transmit the parasite only once (considering that the first bloodmeal was infectious and considering a sporogony lasting 12 days in average). Using the same scenario, control mosquitoes would survive for 27 days in average, and would be infectious through at least three gonotrophic cycles. Moreover, the fecundity of mosquitoes fed at 21 and 28 DAI was significantly reduced by 33 and 20 %, respectively. Mosquitoes weren’t allowed to lay their eggs nor the hatching rate and larvae survival followed, which represent a limitation of this study, probably leading to an under-estimation of the ivermectin treatments effects on mosquito’s fitness. Considering that a mosquito becomes infectious on average 12 days after gametocyte ingestion [39], corrected mortalities [40], i.e., 100 × (% dead mosquitoes fed on treated cattle − % of dead mosquitoes fed on control cattle)/(100 − % dead mosquitoes fed on control cattle) were 75 and 45 % at 21 and 28 days post injection, respectively. For up to 1 month, more than half of the mosquitoes would die before being able to transmit malaria parasites if they were blood fed on treated cattle before or the same day as the infectious blood meal. Moreover, a second ivermectin blood meal at sub-lethal concentrations further increased the mortality, so much that cumulative mosquito mortality was 100 % by day 12 after the second meal.

As previously reported [5, 14, 15, 41], the present study confirms that ivermectin reduces both the life span and fecundity of important and dominant malaria vectors of sub-Saharan Africa feeding on ivermectin-treated hosts. The mosquitocidal effect vanishes at different rates between calves, suggesting a fair variability in the kinetic and dynamic processes of ivermectin distribution, metabolism or clearance, which may impact on the compound availability in peripheral blood vessels. In a recent study, a greater availability of ivermectin was reported in female human volunteers, which has been associated with the greater body mass indices of female by comparison to male participants [15]. Although ivermectin is the less lipophilic of the macrolactones used as antiparasitic compounds, it nevertheless concentrates particularly in adipose tissues, where the limited vascularization and slow turnover rate of fat prolong the residence of the drug in the peripheral blood [32] and, therefore, its availability to vector mosquitoes. The “slow-release reservoir effect” [15] of body fat might also explain why the mosquitocidal effect of ivermectin could last more than a month in this study while in others, this effect disappeared more quickly and is incomplete even shortly after ivermectin administration [5, 15]. Subcutaneous injection distributes a much greater proportion of the ivermectin into lipid reservoirs than oral route and increases its residence time [42]. Moreover, ivermectin maximum concentration (C_max_) is much lower when the drug is administrated by oral route [43], which might further explain the more sustained mosquitocidal effect presented here, but also the greater, complete toxicity of the blood meals taken by *An. coluzzii* on treated cattle, for up to 2 weeks after ivermectin subcutaneous injection. Interestingly, at the sub-lethal doses imbibed by mosquitoes at 31 and 37 days, the deleterious effects of ivermectin on survival diminished after a second blood meal 4 days later, which obviously contained ivermectin at less concentration than the first [10]. A second blood meal that does not contain or contains less ivermectin than the first has been also shown to mitigate the effect of the drug on fecundity and hatch rate in *Aedes aegypti*, *Aedes albopictus* and *Culex quinquefasciatus* [44] and on survival in *An. gambiae s.s* [10]. These cumulative effects must be further examined, considering malaria vectors proclivities for frequent blood feeding in the field [45].

As opposed to in vitro studies [16], the present study failed to demonstrate any transmission blocking properties of sub-lethal concentrations of ivermectin when the ivermectin blood meal was ingested 3 days before an infectious blood meal, but yet at a time post injection where the drug concentration remains toxic enough to impact mosquito survival and fecundity. However, this is in line with recent in situ field studies where ivermectin does not impact gametocyte infectivity [15]. Since only a single DAI (28 days) has been tested on infection prevalence and intensity at the oocyst stage, the present study cannot rule out the possibility that ivermectin has sporontocidal effects. More experiments are needed using different sub-lethal doses, and have to be more adequately designed to study the impact of ivermectin when imbibed with a blood meal at different times after or before the infectious blood meal. However, the present results demonstrate that the stress and corresponding fitness costs induced by sub-lethal doses of the drug did not positively impact infection output, which could have had harmful and counterproductive consequences in terms of transmission, jeopardizing further use of ivermectin to control vector mosquito populations.

The integrative control measure adjunction to existing tools offered by ivermectin has a potential for the management of insecticide resistance since the mode of action of the drug and insecticides currently used for vector control are different. However, despite crucial importance, only a few studies have addressed the question of potential cross-resistance to ivermectin in insecticide-resistant vector mosquitoes. Deus et al. [46] found an increased tolerance to ivermectin imbibed in a blood meal in different *Ae. aegypti* strains resistant to pyrethroid insecticides. For *An. gambiae s.l*., ivermectin decreases mosquito life span of *Anopheles* in different areas of Burkina Faso [14, 15] where the frequency of insecticide target-site mutations, including knock-down resistance (kdr) and insensitive acetylcholinesterase (Ace-1R) alleles, has been regularly monitored and where detoxifying enzymes also contribute to the diversity of resistant phenotypes observed in the field [47, 48]. Thanks to the presence of one out of three mosquitoes carrying the mutated *kdr* allele in the *An. coluzzii* colony used here, this study suggests that no cross-resistance to ivermectin exists in *kdr* carriers, at least at the plasmatic concentrations where mosquitocidal effect is complete. However, proper phenotypic characterization of mutated *kdr* carriers using bioassays would have been needed to actually check the adequacy between the genotype and resistance phenotype. Hence, more studies are definitely needed to decipher this question, knowing the great diversity and complexity of physiological mechanisms allowing wild *An. gambiae s.l.* populations to resist most, if not all, of the insecticide classes used to date as vector control tools [31, 47].

Ivermectin is of capital importance for the control of many parasitic diseases in animals and humans and resistance appearance in endo- or ectoparasitic fauna classically targeted by ivermectin treatments would represent a public health disaster. Ivermectin resistance was reported in small ruminants and cattle nematodes after frequent host treatment [43–45, 49]. Hence, if the “One-Health” approach was to be implemented as an alternative method for the control of malaria vectors, a careful monitoring of potential resistance appearance must be undertaken. Researches must also be prompted to apprehend the risk of an emerging resistance to ivermectin in Anopheles field populations. Indeed, with a much longer mosquitocidal and anti-fecundity effect in cattle serum, longer insecticidal pressure from mosquitocidal cattle blood could select for ivermectin resistance in Anopheles. Recent attempts have been made to better understand the IVM-mosquitoes interactions, where canonical detoxification mechanisms seem to be only marginally involved in the mosquito’s response to ivermectin ingestion, whereas non-canonical pathways are highlighted, notably those involving Nieman–Pick type C-2 family genes [50]. Moreover, the recent discovery of ivermectin sensitive and insensitive glutamate-gated chloride channels generated through alternative splicing, questions this mechanism as the potential target of selection for ivermectin resistance in the field [17]. These findings are important in the sense that they clearly emphasize the complexity of IVM-mosquitoes interactions, which need to be unravelled to better evaluate the risks of emergence of ivermectin resistance in the mosquito populations targeted by ivermectin treatments. Similarly, treating cattle might select for *Anopheles* species/populations with altered behavior toward increased anthropophagy. Hence, the proposed approach would stand only if integrative measures are taken where treatments to humans and cattle and their potential consequences are considered concomitantly. Ivermectin resistance in human or animal targeted parasites and in Anopheles populations are dark shadows in the board of “One-Health” MDAs, and facing these caveats even before they become an issue is the only way to leave this promising approach a reality.

Although subcutaneous administration of ivermectin generates lower faeces concentrations of the product when compared to oral or poor-on formulations [32], non-targeted coprophagic fauna could especially be at risk if MDAs to livestock were implemented [51]. Dung pats are widely used for agricultural purposes in rural Burkina Faso, as everywhere in sub-Saharan rural Africa. Coprophagic fauna accelerate the degradation of dung pats and maintain soil productivity by enhancing the activity of the micro-organisms therein that participate in the mineralization of animal waste. Even sub-lethal doses of ivermectin induce an acute toxicity, altering the sensory and locomotor capacities of dung beetles, and preventing their basic biological activities, ultimately leading to their premature death [52]. However, knowing that sensitivity to ivermectin may vary among species of the same taxa [53], further studies are needed to properly assess ivermectin sensitivity of the coprophagic fauna present in the areas targeted for the “One-health approach”. Such studies are needed so the health benefits to humans and animals of integrated MDAs will not be hampered by potentially high economic losses, which might mitigate the acceptation of this approach by communities.

## Conclusion

This study indicates a sustained, complete effect of ivermectin on the survival of recently colonized *An. coluzzii* females after blood feeding on local calves treated subcutaneously with the recommended therapeutic dose. Moreover, effects of sub-lethal doses are observed on mosquito fecundity, which further increases the impact of ivermectin administrated to cattle on total vector densities. This effect might be even larger than reported here due to the known deleterious effects of sub-lethal doses of ivermectin on mosquito physiology and behaviour, hampering mosquito survival in the field. Further, this study demonstrates the potential of integrated MDAs of ivermectin to both human and peridomestic cattle to target anthropophilic, endophagic, but also exophilic and zoophagic mosquitoes in areas where both physiological and behavioural resistances are widespread, building reservoirs of residual malaria transmission.

At the approved dose of 200 μg/kg used by medical and veterinary services, current oral formulations can only maintain efficient mosquitocidal concentrations for approximately 2–4 days [15], while injected formulations to cattle do so for up to 2 weeks. Interrupting malaria transmission would require a more prolonged mosquitocidal effect [54], which could be obtained either through the distribution of multiple doses of ivermectin, or through the administration of a slow-release formulation, for which research work is on-going [55].

## Additional files

10.1186/s12936-015-1001-z Materials and Method. Survival of *Anopheles coluzzii* females fed once or twice on control or treated cattle with the therapeutic dose of 0.2 mg/kg of Ivermectin.
10.1186/s12936-015-1001-z Mean survival time of Anopheles coluzzii fed once or twice on treated and control cattle at different days after injection (DAI) of 200 µg/kg ivermectin.
10.1186/s12936-015-1001-z Analysis of the effects of ivermectin treatment, the day after injection (DAI), the number of blood meals, and their interaction on female *An. coluzzii*’s survival using the Cox Proportional Hazards model.

## Abbreviations

CIRDES: Centre International de Recherche-Développement sur l’Elevage en zones Subhumides
DAI: days after injection of ivermectin
IRS: indoor residual spraying
IRSS: Institut de Recherche en Sciences de la Santé, Bobo-Dioulasso (Burkina Faso)
LLIN: long-lasting insecticidal nets
MDA: mass drug administration
PBS: phosphate buffered saline
WBC: white blood cells
Kdr: knock down resistance
