# Supplementary material for: Administration of ivermectin to peridomestic cattle: a promising approach to target the residual transmission of human malaria
Source: Malar J. 2015 Dec 10;14:496. doi: 10.1186/s12936-015-1001-z (PMC4676103; doi:10.1186/s12936-015-1001-z)
Supplement: Supplementary file 1 — 10.1186/s12936-015-1001-z Materials and Method. Survival of Anopheles coluzzii females fed once or twice on control or treated cattle with the therapeutic dose of 0.2 mg/kg of Ivermectin. [file 12936_2015_1001_MOESM1_ESM.docx]

Additional file 1. Survival of *Anopheles coluzzii* females fed once or twice on control or treated cattle with the therapeutic dose of 0.2 mg / kg of Ivermectin.

Materials and methods:

Mosquito colony was the same as used in the main experiments (see « Material and Methods »). Mosquito husbandry and handling were identical also, except that the 2.5% glucose solution for maintenance after blood feeding was replaced by a 5% solution. Cattle treatment and maintenance was as described in the main text. At six instances after Ivermectin injection, eight lots of sixty (60) mosquitoes were randomly chosen to be fed on either the treated or control cattle. Different days after injection (DAI) were chosen: 2, 7, 15, 22, 31 and 37 days. Only fully engorged mosquitoes were considered. Thirty percent of these mosquitoes were directly followed for their survival whether for the remaining mosquitoes, a second blood meal was offered on the control or treated cattle 4 days later. Only mosquitoes that had taken 2 consecutive blood meals were subsequently followed for their survival. Survival analyses were performed as described in the main text and results given and commented therein.
