# Supplementary material for: Administration of ivermectin to peridomestic cattle: a promising approach to target the residual transmission of human malaria
Source: Malar J. 2015 Dec 10;14:496. doi: 10.1186/s12936-015-1001-z (PMC4676103; doi:10.1186/s12936-015-1001-z)
Supplement: Supplementary file 2 — 10.1186/s12936-015-1001-z Mean survival time of Anopheles coluzzii fed once or twice on treated and control cattle at different days after injection (DAI) of 200 µg/kg ivermectin. [file 12936_2015_1001_MOESM2_ESM.docx]

Additional file 2 legend: **Mean survival time of *Anopheles coluzzii* fed once or twice on treated and control cattle at different days after injection (DAI) of 200 µg/kg ivermectin.** Black bars: mosquitoes fed once on control calves, light grey bars: mosquitoes fed once on treated calves, dark grey bars: mosquitoes fed twice on control calves, white bars: mosquitoes fed twice on treated calves. Error bars are standard errors.
