# Supplementary material for: Administration of ivermectin to peridomestic cattle: a promising approach to target the residual transmission of human malaria
Source: Malar J. 2015 Dec 10;14:496. doi: 10.1186/s12936-015-1001-z (PMC4676103; doi:10.1186/s12936-015-1001-z)
Supplement: Supplementary file 3 — 10.1186/s12936-015-1001-z Analysis of the effects of ivermectin treatment, the day after injection (DAI), the number of blood meals, and their interaction on female An. coluzzii’s survival using the Cox Proportional Hazards model. [file 12936_2015_1001_MOESM3_ESM.docx]

***Additional file 3.***

Analysis of the effects of ivermectin treatment, the day after injection (DAI), the number of blood meals, and their interaction on female *An. coluzzii* survival using the Cox Proportional Hazards model

| Source | *DF* | Χ^2^ | *P* value |
| --- | --- | --- | --- |
| Treatment | 1 | 467.9223 | <.0001^*^ |
| DAI | 5 | 240.2886 | <.0001^*^ |
| Number of blood meals | 1 | 1.3303 | 0.248 |
| Treatment × DAI | 5 | 274.4006 | <.0001^*^ |
| Treatment × Number of blood meals | 1 | 0.4235 | 0.515 |
| DAI × Number of meals | 5 | 26.8702 | <.0001^*^ |
| Treatment × DAI × Number of meals | 5 | 24.7786 | <.001^*^ |

^*^ Indicates significant effect of parameters or interactions (*P* < 0.05)
